# Study on the relationship between perceived social support and professional identity among young medical professionals from the perspective of New Quality Productive Forces

**DOI:** 10.3389/fpubh.2025.1713656

**Published:** 2025-12-10

**Authors:** Min Zhang, Minrui Li, Zhengyu Chen, Xiaoxin Lin, Kei Un Wong, Hanxiang Gong, Weizhang Huang, Peijun Lin

**Affiliations:** 1 The Second Affiliated Hospital, Guangzhou Medical University, Guangzhou, Guangdong, China; 2 School of Public Health and Primary Care, The Chinese University of Hong Kong, Hong Kong, China; 3 The Fifth Affiliated Hospital, Guangzhou Medical University, Guangzhou, Guangdong, China; 4 Faculty of Humanities and Social Sciences, Macao Polytechnic University, Macao, China; 5 School and Hospital of Stomatology, Guangzhou Medical University, Guangzhou, Guangdong, China

**Keywords:** young medical professionals, professional identity, New Quality Productive Forces (NQPFs), mediating effect, social support

## Abstract

**Background:**

Amidst the rapid reshaping of the healthcare sector by New Quality Productive Forces (NQPFs), an advanced productivity paradigm is characterized by innovation-driven, high-tech, high-efficiency, and high-quality features. This study investigates young medical professionals (18–40 years). As the core workforce driving medical innovation, their professional identity and job autonomy directly influence the development of NQPFs.

**Objective:**

This study aims to explore the relationship between perceived social support and professional identity among young medical professionals within the New Quality Productive Forces (NQPFs) framework, using a parallel mediation model to examine the independent mediating roles of role cognition and work autonomy. The findings are intended to generate hypotheses and provide a theoretical basis for future longitudinal research, which can inform the development of evidence-based policies.

**Methods:**

A total of 730 young professionals (aged 18–40 years) from 12 Grade A Tertiary Hospitals in Guangzhou, Guangdong Province, China, completed online questionnaires. Measurement instruments included Chinese versions of the Professional Identity Scale, Role Cognition Scale, Perceived Social Support Scale, and Work Autonomy Scale. Data analysis involved the use of descriptive statistics with SPSS software and structural equation modeling (SEM) for testing mediating effects via AMOS software.

**Results:**

Perceived social support significantly influenced professional identity, with a total effect of *b* = 0.625. This comprised a direct effect of *b* = 0.374 (accounting for 59.80% of the total effect) and an indirect effect mediated by role cognition of *b* = 0.251. The mediating effect of work autonomy was non-significant.

**Conclusion:**

This study confirmed that perceived social support among young medical professionals directly influences their professional identity, while also indirectly affecting it through role cognition. The mediating role of work autonomy was not substantiated. These findings deepened the understanding of professional identity formation mechanisms in healthcare talent and highlighted the pivotal role of social support and role cognition within the New Quality Productive Forces (NQPFs) context.

## 1 Introduction

Amidst the global transformation and upgrading of healthcare systems, young medical professionals [aged 18–40 years, corresponding to the World Health Organization’s definition of young adults (1)] are confronting unprecedented opportunities and challenges. On one hand, demographic shifts (e.g., aging populations, high prevalence of chronic diseases) (2, 3) and public health crises (such as the COVID-19 pandemic) (4) persistently drive the growth of medical demand, propelling the transition of healthcare service models toward patient-centered, digitalized, and team-based approaches (5). On the other hand, as the core workforce in medicine, these young professionals bear dual responsibilities: inheriting medical knowledge and enhancing healthcare quality, while simultaneously facing multifaceted pressures including occupational stress, career advancement barriers, and professional burnout (6). In recent years, clinical practice and health management research increasingly emphasize the pivotal role of “new-generation medical workers” in healthcare transformation, particularly their professional competence, innovative literacy, and psychological resilience (7). Consequently, deepening the understanding of this group’s occupational psychological characteristics and the mechanisms influencing their professional identity has emerged as a critical imperative in global health management research (8).

New Quality Productive Forces (NQPFs), as an innovative extension of Marxist political economics in the new era, serves as both the theoretical cornerstone of historical materialism in the health industry and the core engine driving the strategic advancement of “Healthy China 2030.” While termed NQPFs in China, this concept aligns with global frameworks such as the Economic Co-operation and Development (OECD)‘s “innovation-driven productivity transformation” (9), the World Bank’s “digital economy development” (10), and Industry 4.0 healthcare modernization initiatives (11). It should be clarified that NQPFs is a theoretical innovation concept with distinct Chinese characteristics recently proposed in China’s academic and policy spheres, yet its core tenets-technological integration, high-quality service delivery, and knowledge-driven growth-resonate with international paradigms for sustainable healthcare development (12). To enhance the international relevance and generalizability of this study, we explicitly position NQPFs within broader global frameworks. The core components of NQPFs-innovation-drive, high-tech integration, and quality enhancement-directly align with established international concepts such as “innovation-driven productivity transformation” (9), “high-quality development” (13), and “digital transformation” in healthcare (14). While the term NQPFs is context-specific, its underlying mechanisms resonate with worldwide efforts to leverage technological advancement for sectoral upgrading (15). Therefore, this study uses the NQPFs framework as a specific instantiation of these universal trends, aiming to generate insights that can inform similar discussions in other contexts undergoing rapid technological and productive shifts. In the healthcare sector, NQPFs manifest prominently in innovative applications of medical technologies (e.g., AI diagnostics, robotic surgery) (16), service model optimization (e.g., precision medicine, telemedicine) (17), are intelligent management system reform (e.g., smart hospital platforms) (18). Although international academia employs similar expressions such as “innovation-driven productivity transformation,” “high-quality development,” or “productivity upgrading” (19), NQPFs as a systematic theoretical framework lacks direct English equivalence. In this paper, the Chinese term xin zhi sheng chan liis translated as ‘New Quality Productive Forces’ (NQPFs) and used consistently hereafter. NQPFs, with technological innovation, factor reconfiguration, and industrial upgrading at its core, provide a novel conceptual framework for transcending traditional development paradigms in healthcare. Its essence lies in achieving a systematic shift from “quantitative accumulation” to “qualitative leap” in medical productivity through iterative upgrades in digital diagnostic technologies, medicine-engineering convergence innovation, health service model restructuring (20). From the perspective of NQPFs perspective, “new-type medical talents” have been endowed with brand-new connotations, that is, they need to possess the ability to apply cutting-edge medical technologies, the ability to integrate interdisciplinary knowledge, and the ability to predict future medical trends. Among them, young medical staff (aged 18–40 years), as a key group that connects the upper and lower levels of the medical system, the dynamic correlation between their sense of social support and professional identity has become an interdisciplinary research topic spanning medical sociology, organizational behavior, and health management.

Internationally, the correlation between social support and professional identity among medical professionals has been empirically validated. Multinational studies demonstrate that robust social support environments significantly alleviate occupational stress, reduce turnover intention, and enhance job satisfaction and professional commitment among healthcare providers (21, 22). Within China’s healthcare system context, however, the construction of social support networks for young medical professionals (aged 18–40 years) faces multidimensional constraints: institutional mechanisms, cultural identity, and societal expectations collectively impact this process. Practical challenges include work-family imbalance (23, 24), and fluctuating social status recognition (25, 26). Amidst the “New Quality Productive Forces (NQPFs)” wave, traditional support systems urgently require adaptation to the rapidly evolving industry ecology. Targeted support for these young professionals is critical to foster their career growth and innovation capabilities. Nevertheless, systematic empirical research on the interaction mechanism between social support and professional identity under the NQPF framework remains limited.

This group of young medical professionals serves not only as practitioners of medical technological innovation but also as the core vehicle for enhancing healthcare quality. Research demonstrates that professional identity, as a pivotal indicator of intrinsic motivation among medical professionals, directly influences their innovation willingness, service quality, and ultimately industry development (27, 28). The refinement of social support networks, deepening of role cognition, and elevation of work autonomy can systematically enhance professional identity levels by mitigating occupational stress and strengthening value perception (25, 26).

However, the current construction of the medical and health talent team still faces structural contradictions, with the coexistence of insufficient total number of innovative talents and inadequate release of the vitality of existing talents (29), manifested through low digital skill penetration, immature interdisciplinary collaboration mechanisms, and singular career development pathways. To provide a robust theoretical foundation, this study integrates several established frameworks. The Job Demands-Resources (JD-R) model (30) offers a macro-level lens, positioning social support and work autonomy as critical job resources that buffer demands and foster positive outcomes like professional identity. Complementing this, Self-Determination Theory (SDT) (31) provides a micro-level explanation, suggesting that social support and autonomy satisfy fundamental psychological needs (for relatedness and autonomy, respectively), which in turn enhance internalization and identity formation. Furthermore, Role Theory (32) elucidates the mediating role of role cognition, positing that clear and coherent role perceptions are essential for reducing ambiguity and strengthening one’s professional self-concept. Finally, the Social Support Buffering Theory (33) specifically frames social support as a mechanism that mitigates the adverse effects of occupational stress on identity.

By integrating these theories, we construct a more rigorous and generalizable model to explain how perceived social support influences professional identity among medical professionals. Based on this, using the perspective of New Quality Productive Forces (NQPFs) as an entry point, this study focuses on exploring the potential mechanisms through which factors such as perceived social support, role cognition, and work autonomy influence professional identity. The objective is to offer more targeted and effective theoretical support and practical guidance for promoting structural balance within the healthcare talent pool, enhancing occupational well-being, driving the development of New Quality Productive Forces in healthcare, and supporting the sustainable development of the medical field.

## 2 Literature review

### 2.1 Research review on medical professionals’ professional identity

Professional identity is a psychological concept referring to an individual’s cognitive alignment between their perspective on a profession’s goals, social value, and other factors, and societal evaluations/expectations of that profession. This kind of identification not only encompasses an individual’s recognition of the nature, content and social value of a profession, but also reflects approval or recognition in multiple aspects such as the identification of professional value, emotional connection with the profession, recognition of professional ability and expectations for career development (21). Furthermore, the significance of interpersonal relationships at work, professional skills, and work autonomy has been increasingly recognized, as they play non-negligible roles in enhancing medical professionals’ professional identity (25). Enhancing professional identity is particularly crucial for addressing the diversification of healthcare demands and increasing occupational stress.

Professional identity among medical professionals serves not only as an intrinsic driver for career development, but also as a critical factor in promoting high-quality healthcare delivery and patient satisfaction. Those with high professional identity typically exhibit stronger capabilities in managing occupational stress and providing superior healthcare services. For medical professionals, interactions with academic colleagues, recognition of their work roles, and their self-perception regarding professional status are pivotal (34). A Peking University survey of clinical students revealed that those with elevated professional identity placed greater emphasis on internalization of professional values, demonstrating significantly enhanced clinical operational competence and patient communication skills compared to low-identity counterparts (35). Research on obstetric medical staff in Suzhou found an inverse correlation between professional identity scores and total work stressors. Meanwhile, organizational commitment indirectly elevated identity levels by strengthening sense of belonging (36). Consequently, understanding and optimizing medical professionals’ professional identity constitutes an essential component of healthcare institution management and policy formulation. In-depth exploration of influencing factors particularly psychosocial determinants beyond material compensation is imperative for constructing an efficient and humanized healthcare delivery system.

### 2.2 Social support theory and professional identity

Social support theory focuses on the material and psychological assistance individuals receive from others and groups within their social environment, and its impact on psychological and behavioral outcomes (33). This theory emphasizes that robust social support networks can provide individuals with emotional sustenance, practical aid, and information resources, thereby mitigating stress, enhancing self-confidence and coping capacity, and promoting physical/psychological well-being and social adaptation. This is consistent with the Social Support Buffering Theory, which posits that support mitigates the negative impact of stressors. It also aligns with the JD-R model, where social support is a key resource, and SDT, where it fulfills the need for relatedness. Introducing social support theory into professional identity research offers new perspectives for examining the formation and development of professional identity among medical professionals. Studies demonstrate that professional identity, as a core driver of individual career development, is intrinsically linked to social support in its formation and maintenance (25, 36). Furthermore, recent research has highlighted the importance of support sources, indicating that different sources of support (e.g., from colleagues, supervisors, family) can have varying impacts on mental health outcomes such as depressive symptoms and loneliness among healthcare employees (37) underscoring the need to consider the multidimensionality of social support. First, emotional support exerts significant effects on professional identity. Understanding, care, and encouragement from colleagues, superiors, and patients strengthen emotional attachment to the profession. When confronting setbacks or occupational stress, emotional support mitigates negative emotions by providing warmth and recognition, thereby reinforcing professional identity. For instance, during diagnostic challenges with complex cases or patient-related misunderstandings, concern from peers and affirmation from supervisors can restore confidence and consolidate career commitment (38). Second, instrumental support demonstrates non-negligible impacts. Encompassing professional training, work guidance, and information sharing, it provides essential scaffolding for enhancing professional competence and occupational literacy. Mentorship accelerates skill acquisition in junior practitioners, boosting competency affirmation; peer exchanges broaden work perspectives and optimize workflow efficiency. These mechanisms consolidate professional foundations while igniting dedication to healthcare delivery (39). Social support operates through multifaceted pathways to elevate medical professionals’ professional identity, establishing itself as a critical optimization factor. Healthcare institutions should actively construct comprehensive support systems by cultivating harmonious work environments, enhancing team cohesion, providing all-round career development support. This elevates professional identity and advances high-quality growth in healthcare services.

From a practical perspective, social support theory provides multidimensional pathways for enhancing professional identity: organizations should establish systems integrating institutionalized support (e.g., transparent career progression pathways) and emotional support (e.g., psychological care mechanisms). Individuals ought to proactively develop social support sources (e.g., joining professional communities). Policy interventions must reinforce social value of occupational groups through industry honors and public recognition. Future research should further explore the role of virtual support in the digital era (e.g., online professional communities), differential impacts of support structures across cultural contexts. This will provide targeted strategies for precision enhancement of professional identity.

### 2.3 Impact of role cognition and work autonomy on professional identity

Role cognition plays a pivotal role in the formation of medical professionals’ professional identity (32). From the perspective of Role Theory, individuals strive for clarity and consistency in their understood roles. When role cognition is high, it reduces role ambiguity and conflict, which are significant job demands in the JD-R model, thereby facilitating a stronger and more positive professional identity. Through clear role cognition, medical professionals gain precise understanding of their responsibilities and goals, which not only enhances job satisfaction but also establishes a firm foundation for developing professional identity (32, 40). When individuals possess explicit comprehension and high recognition of their professional roles, they exhibit stronger inclination to invest emotional commitment and effort toward achieving professional objectives. Furthermore, robust role cognition helps medical professionals maintain occupational stability and self-efficacy when confronting uncertainties and challenges in their careers, thereby reinforcing professional identity (41, 42). This is a central tenet of both the JD-R model, where autonomy is a critical job resource, and SDT, where it directly satisfies the innate psychological need for autonomy, leading to more self-determined motivation and identity integration (31). Consequently, healthcare institutions should prioritize cultivating role cognition during professional development by providing clear career development pathways, deepening comprehension of professional roles. This facilitates the formation and advancement of medical professionals’ professional identity.

Work autonomy serves as a pivotal bridge for enhancing job satisfaction and shaping professional identity in medical professionals’ careers. Within the complex environment of modern healthcare, professionals face unprecedented challenges and pressures, rendering work autonomy not merely instrumental for improving individual work efficacy, but also a significant catalyst for cultivating professional identity. By enhancing work autonomy, medical professionals confronted with escalating job demands and challenges can exercise greater proactivity and creativity. This not only elevates job satisfaction but also establishes foundations for deepening professional identity (43). However, the relationship between work autonomy, social support, and professional identity is neither linear nor unidirectional, but constitutes a dynamic, interactive process (44). This complexity is acknowledged in the JD-R model, which allows for interactions between different resources and demands. Appropriate autonomy can activate intrinsic potential, prompting continuous exploration and recognition of professional roles in practice, thereby deepening commitment to the profession (26, 45). Conversely, excessive autonomy may induce role ambiguity and excessive responsibilities, ultimately undermining job satisfaction and identity formation. Consequently, healthcare institutions play a crucial role. While promoting work autonomy, they must meticulously balance workload and individual capacity. This creates an environment enabling both professional challenge and self-actualization, thereby enhancing healthcare quality and efficiency while simultaneously advancing dual improvement in job satisfaction and professional identity.

### 2.4 Impact of new quality productive forces on professional identity

In today’s rapidly evolving society, New Quality Productive Forces is emerging as a pivotal force driving social progress and economic development (12). From an international perspective, this aligns with the widespread focus on digital transformation and innovation ecosystems as drivers of growth. The impact of NQPFs on professional identity can thus be understood through the lens of how rapid, technology-mediated changes in the workplace affect professionals’ self-perception globally. Human capital constitutes the decisive factor in developing NQPFs. Establishing institutional mechanisms compatible with NQPFs necessitates prioritizing human-centered approaches, further deepening structural reforms to accelerate talent cultivation and enhance human capabilities (20). Characterized by deep integration of digital technologies, iterative innovation in diagnostic-therapeutic models, and construction of green-smart healthcare ecosystems, NQPFs is fundamentally reshaping the professional connotation and value systems of the healthcare industry, thereby exerting multidimensional impacts on medical professionals’ professional identity (46). Professional identity, as the subjective recognition of medical staff towards their professional mission, professional capabilities and development paths, is closely intertwined with the technological empowerment driven by NQPFs, the transformation of service models and the upgrading of health governance during its construction process. At the technological application level, the proliferation of digital tools (e.g., AI-assisted diagnostic systems, blockchain-based electronic health records, remote surgical robots) facilitates the transition of medical professionals from executors of traditional clinical workflows to “intelligent healthcare decision-making collaborators.” Through enhanced human-machine collaboration efficiency and precision medicine practices (47), this reinforces their identification with technical adaptability and professional indispensability. This phenomenon is not unique to China; it reflects a global trend where digital transformation compels professionals to adapt their roles, impacting identity formation in various national contexts. At the industrial ecology level, emerging fields catalyzed by NQPFs (e.g., internet healthcare, precision medicine, regenerative medicine) endow medical professions with heightened scientific-social value (48). For instance, clinicians engaged in AI-driven cancer early-screening R&D perceive strengthened alignment between their professional value and the Healthy China 2030 strategy, prompting deep binding of personal career development to national health objectives and intensifying identification with medicine’s societal significance (49). This underscores a universal mechanism: when national or institutional priorities for high-quality development align with professional activities, they can significantly strengthen professional identity. At the organizational transformation level, Flexible diagnostic-therapeutic units within smart hospital frameworks, such as multidisciplinary consultation platforms, cloud-based MDT teams, and cross-institutional clinical data-sharing platforms-dissolve traditional departmental barriers. These create more innovative and autonomous work scenarios, enabling medical professionals to lead integrated “diagnosis-research-technology translation” practices. This drives their role reconstruction from passive executors to proactive value creators, strengthening emotional belonging and role identity (50).

Empirical studies indicate that medical professionals in institutions with mature NQPFs applications exhibit higher composite scores in professional identity, with particularly significant improvements in the dimensions of “occupational innovation value” and “social contribution expectations”(51). Surveys from smart hospitals demonstrate that physicians participating in 5G-enabled telemedicine projects experience enhanced occupational meaningfulness, as they directly witness technology’s empowerment of diagnostic capabilities in remote areas and effective reduction of emergency response time and are more inclined to incorporate “technology-driven healthcare equity” into their professional objectives (52). However, pressures from technological iteration (e.g., prolonged adaptation cycles for digital tools among physicians over 60) and responsibility reconstruction (e.g., ethical attribution controversies in AI misdiagnosis) may induce professional identity confusion in some practitioners (53). Consequently, healthcare institutions must explore the construction of tiered digital literacy training systems, human-machine collaboration ethics guidelines and innovative diagnostic-therapeutic error-tolerance mechanisms. For instance, establishing AI-assisted diagnosis training bases and intelligent medical ethics committees can effectively alleviate transitional disruptions and promote positive evolution of professional identity.

Furthermore, NQPFs is inherently embedded within the interaction between productive forces and production relations (20). The adaptive transformation of social support systems becomes pivotal for mitigating resultant tensions. Healthcare institutions can reinforce the synergistic effects of technological empowerment and humanistic care by establishing a trinity support network encompassing technical training, psychological support, career development. At the policy level, it is necessary to balance technological innovation and humanistic values. On the one hand, medical data security regulations should be improved to safeguard professional dignity, and on the other hand, a technical ethics review mechanism should be established to prevent algorithmic bias from eroding professional fairness (54).

Collectively, the interplay between NQPFs and medical professionals’ professional identity manifests as a dynamic process of “technological empowerment-value reconstruction-institutional adaptation.” From an industry development perspective, the shaping of professional identity by NQPFs constitutes a synergistic innovation integrating medical technological advancement with professional ethos inheritance. Future research can further focus on the virtual construction of professional identities in the metaverse medical scenario, the impact of AI medical assistants on the professional boundaries of doctors, and the identification formation mechanism of “AI native generation “medical staff (post-95 s practitioners) in the intelligent diagnosis and treatment environment, providing theoretical support for optimizing the medical talent training system and building a human-machine collaborative career development ecosystem (55, 56). Build a resilient framework for professional identity to help medical staff transform from “tool adaptors” to “value leaders” in the technological wave.

While the broader context of New Quality Productive Forces (NQPFs) shapes the operational environment for healthcare professionals, this study focuses specifically on the psychological mechanisms through which perceived social support influences professional identity. Prior research has established correlations between social support and positive occupational outcomes, but the mediating pathways, particularly the parallel roles of role cognition and work autonomy, remain less explored within the medical profession. Understanding these mechanisms is crucial for developing targeted interventions.

Based on the integrated theoretical framework of JD-R, SDT, Role Theory, and Social Support Buffering Theory, this study aims to test a parallel mediation model. We hypothesize that perceived social support positively influences the professional identity of young medical professionals. Furthermore, we propose that this relationship is mediated by two distinct pathways:

*H1*: Perceived social support will have a direct positive effect on professional identity.

*H2*: Role cognition will mediate the relationship between perceived social support and professional identity. Specifically, higher perceived social support will be associated with clearer role cognition, which in turn will lead to stronger professional identity.

**Figure 1:**
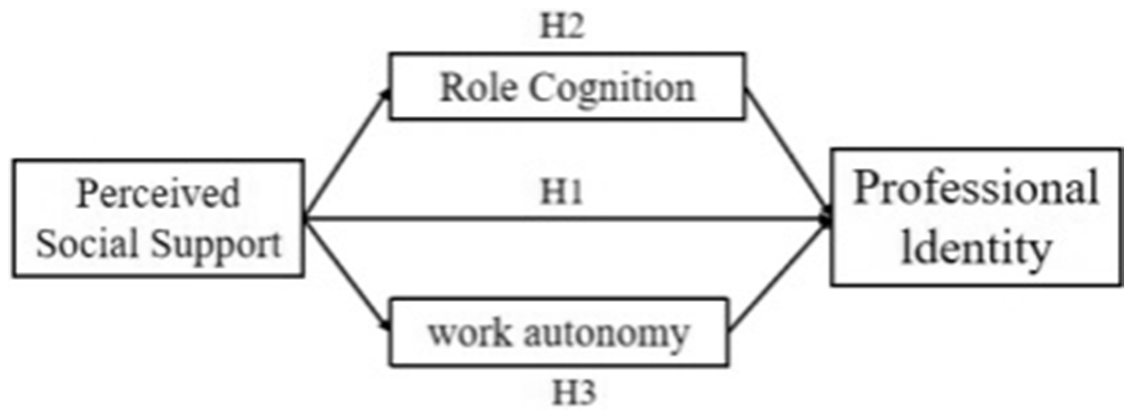
Parallel mediation model hypothesis diagram.

*H3*: Work autonomy will mediate the relationship between perceived social support and professional identity. Specifically, higher perceived social support will be associated with greater perceived work autonomy, which in turn will lead to stronger professional identity (see Figure 1).

By testing these hypotheses, this study seeks to elucidate the psychological processes underlying the social support-professional identity link, providing a more nuanced understanding that can inform support strategies for medical professionals beyond the overarching NQPF context.

## 3 Materials and methods

### 3.1 Study subjects

A cross-sectional study was conducted by surveying medical professionals from municipal hospitals in Guangzhou during July–August 2023. Electronic questionnaires were administered using the Questionnaire Star online platform (wjx.cn) via QR code distribution. The first page of the questionnaire presented informed consent information, and participants could voluntarily exit if they declined participation. Measurement instruments included the Chinese versions of: the Professional Identity Scale (57), the Role Cognition Scale (58), the Perceived Social Support Scale (59), and the Work Autonomy Scale (60). The study protocol was approved by the Clinical Research and Application Ethics Committee of the Second Affiliated Hospital of Guangzhou Medical University. Inclusion criteria: (1) healthcare workers aged 18–40 years (physicians, nurses, medical technicians, etc.); (2) voluntary participation. Exclusion criteria: (1) administrative or clerical staff not directly engaged in clinical practice; (2) individuals declining participation or unable to cooperate. Questionnaires with identical responses throughout or completion time <100 s were deemed invalid. A total of 730 questionnaires were collected, with invalid responses excluded, yielding an effective response rate of 98.77% (*n* = 721). Reliability and validity of the questionnaire were assessed using Cronbach’s *α* coefficient, Kaiser–Meyer–Olkin (KMO) measure, and Bartlett’s test of sphericity. Results indicated Cronbach’s *α* > 0.9 and KMO > 0.9 (both *p* < 0.001), confirming robust psychometric properties and supporting further analytical validation.

### 3.2 Research methods

#### 3.2.1 General information form

Survey indicators encompassed personal information including occupation, gender, age, and educational background.

#### 3.2.2 Professional identity scale

Professional identity refers to individuals’ confidence in their work, endorsement of professional life, and adaptation to and affirmation of their occupation; comprising 12 items across dimensions of work identity recognition, recognition from others, and Recognition of work. The scale demonstrated a Cronbach’s *α* coefficient of 0.918 in this study.

#### 3.2.3 Role cognition scale

Containing 16 items across two dimensions: medical professionals’ cognition of role-required competencies and manifested humanistic care philosophy. The scale exhibited a Cronbach’s *α* coefficient of 0.975 in this study.

#### 3.2.4 Perceived Social Support Scale

Comprising 12 items measuring individuals’ subjectively perceived social support levels, categorized into familial support and external support. The scale achieved a Cronbach’s *α* coefficient of 0.950 in this study.

#### 3.2.5 Work autonomy scale

Incorporating 6 items assessing individuals’ control or discretion over work methodology autonomy, work scheduling autonomy, and performance standard autonomy. The scale yielded a Cronbach’s α coefficient of 0.935 in this study. All questionnaires utilized a five-point Likert scale ranging from 1 (Strongly Disagree) to 5 (Strongly Agree), with scores 1–5 assigned accordingly. Responses ≥3 were defined as endorsement, otherwise as non-endorsement. Higher scores indicated stronger agreement with item content.

### 3.3 Statistical methods

A two-step procedure involving confirmatory factor analysis (CFA) and structural equation modeling (61, 62) was employed to test the hypotheses. In the first step, we conducted CFA to examine the distinctiveness of the variables used in the study. In the second step. We used a Parallel Mediation Model to evaluate our structural models. Statistical analyses were performed using SPSS 25.0 and AMOS 26.0. General demographic characteristics were analyzed as categorical data; correlation analyses employed Spearman’s test. A structured equation model of mediating effect was established using Amos 26.0 to analyze the influence relationship of three observed variables, namely the social support, role cognition and work autonomy of medical staff, on professional identity. A two-tailed test significance level of *α* = 0.05 was applied.

## 4 Results and analysis

### 4.1 Demographic characteristics of the surveyed population

Among the 721 people surveyed, the number of doctors and nurses was close, at 337 and 309, respectively, (accounting for 46.74 and 42.86%), the remaining 75 participants accounting for 10.4% (medical technicians, pharmaceutical personnel, and administrative/logistics staff). The number of men and women was 208 and 513, respectively, (accounting for 28.85 and 71.15%). There are 537 people (74.48%) with a bachelor’s degree or below and 184 people (25.52%) with a master’s degree or above. The number of people with intermediate or lower professional titles was at most 543 (75.31%), and that of those with associate senior professional titles or above was 178 (24.69%). The highest monthly income was below 10,000 yuan, with a total of 390 people (54.09%). The number of people with 10 years or less of working experience and those with more than 10 years of working experience were 309 and 412, respectively, (accounting for 42.86 and 51.74%).

### 4.2 CFA results

At the same time, to determine whether each item can effectively represent the latent variable it is intended to measure, factor loadings are used for judgment. To assess model fit, we used the overall model chi-square measure (c2), the Tucker–Lewis Index (63), the comparative fit index (64), and the root mean square error of approximation (65) (see Table 1).

**Table 1:** Presents the CFA results.

Model	The range of factor loadings	*χ*^2^	df	*χ*^2^/df	CFI	TLI	RMSEA
4-Factor model (perceived social support, role cognition, work autonomy, professional identity)	0.457~0.941	4803.126	970	4.952	0.891	0.884	0.074
3-Factor model (perceived social support, role cognition + work autonomy, professional identity)	0.470~0.900	2928.736	399	7.340	0.882	0.871	0.094
2-Factor model (perceived social support + work autonomy + role cognition, professional identity)	0.473~0.891	3483.667	251	13.879	0.815	0.797	0.134
1-Factor model (perceived social support + role cognition + work autonomy + professional identity)		32600.007	1,035	34.976	1.000	–	0.217

As shown in that table, the fit indices revealed support for the hypothesized 4-factor model suggesting support for the distinctiveness of the constructs used in this study. The lowest factor loading of each item is greater than 0.30, which can effectively represent the latent variable it measures (74).

### 4.3 Factor analysis

Varimax rotation was employed for factor analysis of four scales. Common factors were retained based on: (1) eigenvalues (*λ*) ≥ 1; (2) Cumulative variance contribution rate of *k* common factors exceeding 70%.

#### 4.3.1 Quantitative factor analysis of professional identity

Three factors were extracted, explaining 71.856% of total variance: Items 1–4 loaded on Factor 1 (Work identity recognition). Items 5, 8 loaded on Factor 2 (recognition from others). Items 6, 7, 9–12 loaded on Factor 3 (Recognition of work). Details see Table 2.

**Table 2:** Factor analysis results of professional identity scale.

Content	Factor loadings (post-rotation)			Communality
	Factor 1	Factor 2	Factor 3	
A1 When discussing my profession, I typically say “we” rather than “they”	0.382	0.636		0.559
A2 Professional success of medical practitioners is my success	0.279	0.786		0.697
A3 I care about others’ views on my profession		0.667	0.408	0.615
A4 Praise for my profession feels like personal praise	0.164	0.777	0.310	0.727
A5 Media criticism of my profession provokes strong indignation	0.271	0.318	0.741	0.724
A6 My work is significant and meaningful	0.626	0.307	0.512	0.748
A7 I am confident in my professional competence	0.751	0.229	0.361	0.746
A8 My work influences patients’ conditions	0.450	0.129	0.667	0.664
A9My academic specialization facilitates work execution	0.772	0.180	0.381	0.774
A10 I understand work content and requirements	0.821	0.149	0.367	0.830
A11 Medical work aligns with my professional suitability	0.754	0.377		0.714
A12 I comprehend my professional role	0.865	0.230	0.154	0.824
Eigenvalues (post-rotation)	4.071	2.585	1.966	
Variance explained (%) (post-rotation)	33.929	21.543	16.384	
Cumulative variance (%) (post-rotation)	33.929	55.472	71.856	

#### 4.3.2 Factor analysis of Perceived Social Support, Role Cognition, and Work Autonomy Scales

Perceived Social Support Scale extracted two factors explaining 74.954% of total variance: Items 3, 4, 8, 10 loaded on Factor 1 (familial support), Remaining items loaded on Factor 2 (external support). Role Cognition Scale extracted two factors explaining 79.854% of total variance: Items 2, 7–9, 14–15 loaded on Factor 1 (compassionate identity), remaining items loaded on Factor 2 (professional identity). Work Autonomy Scale extracted two factors explaining 83.984% of total variance: Items 1–5 loaded on Factor 1 (work autonomy), Remaining items loaded on Factor 2 (life autonomy).

### 4.4 Descriptive statistics and correlation analysis of perceived social support, role cognition, work autonomy, and professional identity among young medical professionals

The normality of the data for perceived social support, role cognition, work autonomy, and professional identity was assessed using the Kolmogorov–Smirnov (K–S test) test. Results indicated that the data for all scales significantly deviated from a normal distribution (all *p* < 0.001). Therefore, the median (Med) and inter quartile range (IQR) were used to describe central tendency and dispersion.

The extent of dispersion was evaluated based on the ratio of the IQR to the total range (Max − Min). A larger ratio suggests that the middle portion of the data is relatively spread out, indicating high dispersion; a smaller ratio reflects greater concentration in the central data, indicating low dispersion. As shown in Table 3, scores on most scales were generally above 3 (on a 5-point scale), and the IQR accounted for a relatively small proportion of the total range, indicating low overall dispersion. This pattern suggests that most medical staff reported relatively high levels of identification in terms of role cognition and professional identity, while their levels of perceived social support and work autonomy were moderately positive but not particularly high. Pearson correlation analysis revealed significant positive correlations among all four variables (*p* < 0.001 for all coefficients).

**Table 3:** Descriptive statistics and correlation analysis of perceived social support, role cognition, work autonomy, and professional identity.

Scale	Med	IQR (Q3–Q1)	Range	Perceived social support	Role cognition	Work autonomy	Professional identity
Perceived social support	3.58	1	4	1			
Role cognition	4.69	1	4	0.471*	1		
Work autonomy	3	1	4	0.536*	0.309*	1	
Professional identity	4	0.92	4	0.590*	0.628*	0.350*	1

*p < 0.001.

### 4.5 Mediating effect analysis

#### 4.5.1 Model fit indices

A parallel mediation model was established with perceived social support as the independent variable, role cognition and work autonomy as mediators, and professional identity as the dependent variable. Initial model fit indices indicated poor fit: *χ*^2^/df = 13.126, RMSEA = 0.130, CFI = 0.936, GFI = 0.917, TLI = 0.896. This inadequacy primarily stemmed from the non-significant indirect effect of work autonomy on professional identity (*β* = −0.003, *p* = 0.902). After removing work autonomy, the modification indices suggested adding covariance relationships between the error terms of several observed variables (e1–e5, e1–e4, e2–e6, e4–e7, e5–e8). These modifications were not arbitrary but were implemented based on strong substantive and methodological rationales to achieve a more parsimonious and theoretically sound measurement model (66, 67). The decision to allow these error covariances was based on two primary considerations: (1) Shared Method Variance: The scales utilized a common method (self-report Likert-scale questionnaires), which can introduce systematic covariance not accounted for by the latent constructs themselves. Correlating errors within the same scale (e.g., within the Perceived Social Support Scale) helps to control for this common method bias, leading to more accurate estimates of the structural relationships (68). (2) Substantive overlap in item content: some sections of the different questionnaires contain conceptual overlap or highly similar phrasing that could cause their error terms to correlate beyond the influence of the overarching latent variable. For instance, the correlation between errors e1 and e4 might reflect a shared specific nuance in measuring a facet of support that is not fully captured by the broader “familial support” factor. This approach is a recognized and justified practice in Structural Equation Modeling (SEM) to improve model fit when the modifications align with theoretical expectations about the measurement instrument (69).

It is important to note that these modifications were applied to the measurement model (i.e., the relationships between indicators and their latent constructs) and did not alter the hypothesized structural model (the paths between latent variables). Therefore, they enhance the validity of the measurement without capitalizing on chance relationships in the structural paths, thus mitigating concerns of overfitting the structural model (70). Post-modification fit indices met all criteria (Table 4), confirming acceptable model fit.

**Table 4:** Model fit indices.

Fit index	*χ*^2^	df	*χ*^2^/df	GFI	TLI	CFI	NFI	RMSEA
Criterion	–	–	1–3	>0.9	>0.9	>0.9	>0.9	<0.08
Value	17.822	6	2.970	0.993	0.987	0.996	0.995	0.052

#### 4.5.2 Bootstrap testing

Using Bootstrap sampling with 5,000 repetitions, total path effects were 0.625 (indirect mediating effect = 0.251; direct effect = 0.374). The indirect and direct effects accounted for 40.20 and 59.80% of total effects, respectively. All 95% CIs excluded zero (*Z* > 1.96, *p* < 0.001), confirming role cognition’s significant partial mediating effect (Table 5).

**Table 5:** Bootstrap analysis of path effect significance.

Path	Effect type	Point estimate	Product of coefficients		95% CI	Proportion of total effect (%)	*p*
			SE	*Z*			
Perceived social support → role cognition → professional identity	Indirect mediating	0.251	0.035	7.171	0.197 ~ 0.310	40.20	<0.001
Perceived social support → professional identity	Direct	0.374	0.044	8.500	0.304 ~ 0.451	59.80	<0.001
Perceived social support → professional identity	Total	0.625	0.047	13.298	0.550 ~ 0.703	–	<0.001

#### 4.5.3 Mediation model

**Figure 2:**
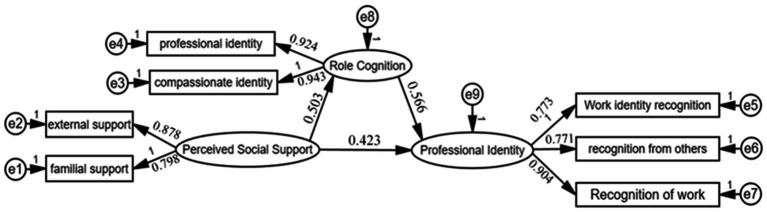
Mediation model of role cognition between perceived social support and professional identity among medical professionals.

A mediation model was established to verify that perceived social support exerted a positive effect on professional identity (direct effect coefficient = 0.423); perceived social support positively influenced role cognition (direct effect coefficient = 0.503); and role cognition positively affected professional identity (direct effect coefficient = 0.566). All path coefficients were statistically significant (*p* < 0.001) (Table 6; Figure 2).

**Table 6:** Effects of perceived social support and role cognition on professional identity.

Path	Standardized path coefficient	Unstandardized path coefficient	SE	*T*-value	*p*-value
Perceived social support → role cognition	0.503	0.444	0.036	12.435	<0.001
Role cognition → professional identity	0.566	0.566	0.049	11.580	<0.001
Perceived social support → professional identity	0.423	0.374	0.034	10.876	<0.001

## 5 Discussion

### 5.1 Structural equilibrium of healthcare talent deployment requires optimization

The development of new-quality productive forces in the medical field invariably originates from “innovation” as the conceptual genesis, centers on “quality enhancement,” and ultimately manifests as “productive output.” This trajectory mirrors the core objectives of innovation-driven growth and high-quality development strategies observed in health systems worldwide. Talent constitutes the primary element of innovation, wherein only innovation can yield substantive achievements and breakthroughs. Structural imbalances identified here parallel challenges in Europe and North America, where rural–urban disparities and digital skill gaps hinder equitable care (e.g., OECD Health Statistics 2023). The current study revealed young medical professionals’ professional identity median at 4 and perceived social support at 3.58. Measured against the maximum score of 5 on the Likert five-point scale, these metrics indicate significant potential for enhancement. While physician and nurse populations were comparable in size, gender structure skewed predominantly female (72.4%), educational attainment focused on bachelor’s degree or lower (89.1%), professionals with Associate Senior Title or above remained scarce (18.3%), and most reported compensation levels below ¥10,000 monthly (76.8%). Concurrently, the cohort universally endured dual pressures from familial obligations and occupational demands, while confronting promotion barriers and relatively inadequate remuneration. This evidence substantiates that structural imbalances within healthcare talent deployment extend beyond quantitative gaps between elite innovators and routine practitioners, fundamentally reflecting inequitable distribution across three critical dimensions: policy support systems, resource allocation mechanisms, and incentive structures. Scholars including Wang and Tang (36) have corroborated this phenomenon.

In conclusion, the relatively moderate levels of professional identity and social support found in this study, coupled with the demographic profile of the sample (e.g., high proportion of early-career professionals with lower titles and compensation), the moderate scores on key psychological constructs suggest that the current “static allocation” of resources may not be effectively supporting the workforce’s professional identity. Provide empirical grounding for the theoretical argument that a paradigm shift is needed.

### 5.2 The marginal mediating role of work autonomy

Against the backdrop of new-quality productive forces driving healthcare transformation, work autonomy has emerged as a crucial indicator gauging talent innovation vitality and career development quality. It functions not only as an endogenous driver for activating individual potential and enhancing diagnostic-therapeutic efficiency, but also constitutes a fundamental psychological foundation for building professional identity. The current study found young medical professionals’ work autonomy score at 3.09 ± 0.92, corresponding to the “moderate” level on the measurement scale. Notably, while significant positive correlations existed among work autonomy, professional identity, perceived social support, and role cognition, these correlations dissipated upon incorporating work autonomy into variance-based modeling approaches. Contrary to expectations based on Self-Determination Theory (31), work autonomy did not significantly mediate the social support-professional identity relationship. This non-finding may reflect the unique context of healthcare organizations implementing NQPF technologies. Rather than experiencing autonomy as empowering, medical professionals may perceive it as “burdensome responsibility” when coupled with high accountability and complex decision-making requirements (71). In rapidly evolving healthcare systems, autonomy without adequate structural support may increase perceived demands rather than resources. This interpretation aligns with the JD-R model’s premise that job characteristics can function as either resources or demands depending on context (71). Our results suggest that in Chinese tertiary hospitals, the current implementation of work autonomy may not provide the psychological need satisfaction predicted by SDT. Specifically, within the context of public welfare-oriented reforms in hospitals, medical professionals universally confront dual pressures of cost containment and service quality enhancement. Coupled with rigid management hierarchies and insufficient equipment/technical support, these elements collectively curtail work autonomy, particularly in scenarios requiring strict adherence to established protocols.

### 5.3 The significant indirect mediating effect of role cognition between perceived social support and professional identity

Medical professionals serve not only as direct providers of healthcare services, but also as core forces driving high-quality development of healthcare systems and achieving the strategic objectives of Healthy China. Research demonstrates that role cognition—defined as practitioners’ awareness of requisite professional competencies and embodiment of humanistic care principles in clinical practice—exerts a significant indirect mediating effect in the influence pathway from perceived social support to professional identity. The significant mediating effect of role cognition (*b* = 0.251) aligns with Role Theory and the JD-R model. This suggests that social support enhances professional identity primarily by helping medical professionals clarify their roles and responsibilities. When healthcare workers receive adequate support, they develop clearer understanding of their professional expectations, which reduces role ambiguity and strengthens identity formation. This finding corroborates previous research indicating that role clarity is a critical mechanism through which organizational resources influence employee outcomes (32, 40). In high-demand healthcare environments characteristic of NQPF development, role cognition may be particularly crucial for helping professionals navigate complex responsibilities and technological changes.

Perceived social support, as a critical psychological resource within organizational environments, serves as a fundamental guarantee for activating medical professionals’ innovative vitality and fostering occupational belonging. Its manifestation extends beyond improvements in working conditions and compensation enhancement, fundamentally residing in organizational culture’s respect and recognition of individual value. Robust perceived social support effectively mitigates professional burnout among medical professionals while enhancing their psychological resilience, thereby catalyzing heightened initiative and creativity in clinical practice. This “soft power” synergizes with the “hard power” epitomized by role cognition, collectively constituting the cornerstone of medical professionals’ professional identity.

Further strengthening perceived social support and deepening medical professionals’ awareness of their role competence facilitates the construction of a more human-centric, synergistically efficient healthcare ecosystem. This system not only encourages continuous advancement of specialized skills but also provides platforms for innovative practice. In emerging domains such as intelligent healthcare systems, telemedicine platforms, and precision medicine initiatives, medical professionals’ proactive engagement and technological exploration will emerge as the key propelling force driving transformative shifts in healthcare models.

### 5.4 The direct effect of social support on professional identity

Findings reveal that perceived social support exerts a significant direct effect on the professional identity of medical professionals (*b* = 0.374), accounting for 59.8% of the total effect. This finding aligns with empirical studies by Zhao et al. (22), and Luo et al. (25), confirming the fundamental role of social support in professional identity formation. Theoretically, the direct effect reflects immediate reinforcement through emotional resonance and value validation (33). Specifically, support from four dimensions—familial, collegial, supervisory, and organizational-constitutes a multidimensional support network wherein familial support provides emotional security, collegial support enhances belongingness, supervisory support offers value recognition, and organizational support creates institutional safeguards. This multidimensional system significantly strengthens professional identity by satisfying medical professionals’ relatedness needs and competence needs (31).

Furthermore, descriptive statistical analysis indicates that the median score of perceived social support among young medical professionals is 3.58 (on a 5-point scale), reflecting an upper-moderate level with room for improvement. This result suggests that while preliminary progress has been made in constructing support systems within healthcare environments, a gap remains from the ideal state. Specifically, medical professionals still face significant pressures in career advancement systems (e.g., professional title evaluation channels), income distribution mechanisms, organizational climate, and social recognition. Some practitioners experience occupational burnout, diminished work motivation, and insufficient social status recognition, echoing findings from Kuhlmann et al. (72) regarding healthcare workers’ mental health.

Compared with European and American healthcare systems, China’s social support system for medical professionals shows evident deficiencies in systematicity and diversity. These gaps mainly manifest in three aspects: first, singular support sources-overreliance on workplace support with underdeveloped community and societal-level support networks; second, traditional support methods-failure to fully utilize digital means to construct new support platforms; third, fragmented support content—lack of stratified support strategies tailored to different career stages of medical professionals (36). These systemic deficiencies constrain the development of medical professionals’ professional identity and the release of occupational vitality to some extent, providing theoretical basis for future optimization directions.

## 6 Conclusion

This study, grounded in the framework of New Quality Productive Forces (NQPFs), our findings partially support the integrated theoretical framework presented in the introduction. The significant role of role cognition emphasizes the importance of cognitive clarity in identity formation, consistent with Role Theory. However, the non-significant autonomy pathway challenges straightforward application of SDT in this specific healthcare context. This discrepancy may reflect the unique demands of medical practice during periods of rapid technological transformation under NQPF initiatives. Compared to previous research, our results both converge and diverge from existing literature. The mediating role of role cognition supports findings by Yang et al. (42), and Wang et al. (41) regarding the importance of role clarity in healthcare settings. However, the non-significant autonomy effect contrasts with studies conducted in Western healthcare contexts (73), suggesting potential cultural or organizational differences in how autonomy operates within Chinese medical institutions. This highlights the need for context-sensitive theoretical applications when studying healthcare professionals in different cultural and organizational environments.

As with any research, the present one has a number of limitations. A First limitation is the cross-sectional design prohibits definitive causal inferences about the relationships between perceived social support, role cognition, and professional identity. While our model is theoretically grounded, the temporal sequence of these variables cannot be established. Future studies should adopt longitudinal or experimental designs to corroborate the causal pathways identified here.

A second limitation is the data were collected exclusively from young medical professionals in Grade A Tertiary Hospitals in Guangzhou. This limits the generalizability of the findings to other regions, different levels of healthcare institutions (e.g., community health centers), or other age groups of medical staff. Replicating this study with a more diverse and nationally representative sample would enhance the external validity of the findings.

A third limitation is the use of self-report questionnaires, while practical, carries the risk of common method bias and social desirability bias. Although we statistically controlled for some common method variance through model modifications, future research would benefit from employing multi-source data (e.g., supervisor ratings of performance, objective indicators of innovation) or more objective measures to validate the constructs.

A fourth limitation is the non-significant mediating role of work autonomy was an important finding, but the reasons behind it warrant deeper investigation. Qualitative inquiries, such as in-depth interviews, could be particularly valuable for exploring the contextual factors (e.g., specific hospital policies, leadership styles) that shape how autonomy is perceived—either as a resource or a demand—in different healthcare settings.

These limitations notwithstanding, the present findings provide support for the generalizability of perceived social support theory to the healthcare sector within the innovation-driven context of New Quality Productive Forces (NQPFs). Specifically, it replicated the demonstrated influence of perceived social support on the professional identity of young medical professionals, with a total effect of 0.625 comprising a direct effect (0.374) and an indirect effect mediated by role cognition (0.251). Further and more importantly, the findings provide an explanation for why perceived social support is related to professional identity: the significant mediating role of role cognition highlights the cognitive and psychological underpinnings of this relationship, wherein social support enhances clarity in role understanding, thereby fostering identity formation. Conversely, the non-significant mediating role of work autonomy suggests that in the current high-demand healthcare environment, autonomy may be perceived as a burden rather than a resource, limiting its positive impact. Lastly, the findings support the role of perceived social support as an antecedent of role cognition. The extent to which medical professionals perceive support from their organization, colleagues, and society promotes trust in their roles and provides the “experiential base for the development of professional identity.”

To conclude, the present study provides an explanation for why perceived social support has been shown to be related to important professional outcomes such as enhanced professional identity and innovation willingness within the NQPFs framework. Although previous research has examined social support as an antecedent and mediator, there is a dearth of research that has examined why it is related to professional identity in the context of rapid technological and productive shifts driven by NQPFs. Given the increasing interest in the healthcare professional-organization relationship in a changing medical context characterized by digital transformation and quality enhancement, it is hoped that the present study will provide an impetus for research that tests social support theory particularly in cross-cultural and interdisciplinary settings, aligning with global frameworks such as innovation-driven productivity and high-quality development.

## Data Availability

The raw data supporting the conclusions of this article will be made available by the authors, without undue reservation.
